# Two Novel Antihypertensive Peptides Identified in Millet Bran Glutelin-2 Hydrolysates: Purification, In Silico Characterization, Molecular Docking with ACE and Stability in Various Food Processing Conditions

**DOI:** 10.3390/foods11091355

**Published:** 2022-05-06

**Authors:** Yajun Zheng, Xueying Wang, Min Guo, Xiaoting Yan, Yongliang Zhuang, Yue Sun, Junru Li

**Affiliations:** 1 College of Food Science, Shanxi Normal University, Taiyuan 030092, China; wxy18835739022@163.com (X.W.); gm13754838917@163.com (M.G.); t113355a@163.com (X.Y.); sy18834288080@163.com (Y.S.); lijunru_1@163.com (J.L.); 2 Yunnan Institute of Food Safety, Kunming University of Science and Technology, Kunming 650500, China; kmylzhuang@163.com

**Keywords:** millet bran glutelin-2 hydrolysates, angiotensin-I converting enzyme inhibitor, molecular docking, stability, security prediction in silico

## Abstract

The addition of food-derived antihypertensive peptides to the diet is considered a reasonable antihypertension strategy. However, data about the stability of antihypertensive peptides in different food processing conditions are limited. In this study, through Sephadex G-15 gel chromatography and RP-HPLC separation, UPLC–ESI–MS/MS analysis and in silico screening, two novel ACE-inhibitory peptides, Pro-Leu-Leu-Lys (IC_50_: 549.87 μmol/L) and Pro-Pro-Met-Trp-Pro-Phe-Val (IC_50_: 364.62 μmol/L), were identified in millet bran glutelin-2 hydrolysates. The inhibition of angiotensin-I converting enzyme and the potential safety of PLLK and PPMWPFV were studied using molecular docking and in silico prediction, respectively. The results demonstrated that PLLK and PPMWPFV could non-competitively bind to one and seven binding sites of ACE through short hydrogen bonds, respectively. Both PLLK and PPMWPFV were resistant to different pH values (2.0–10.0), pasteurization conditions, addition of Na^+^, Mg^2+^ or K^+^ and simulated gastrointestinal digestion. However, PLLK and PPMWPFV were unstable upon heat treatment at 100 °C for more than 20 min or treatment with Fe^3+^ or Zn^2+^. In fact, treatment with Fe^3+^ or Zn^2+^ induced the formation of PLLK–iron or PLLK–zinc chelates and reduced the ACE-inhibitory activity of PLLK. These results indicate that peptides derived from millet bran could be added to foods as antihypertension agents.

## 1. Introduction

In 2021, around 1.28 billion people were suffering from hypertension and its complications such as arteriosclerosis, hypertensive encephalopathy, stroke and myocardial infarction [1]. More than one-sixth of the antihypertensive drugs used in the world are angiotensin I-converting enzyme (ACE) inhibitors, because ACE is a crucial enzyme in the processes that elevate human blood pressure [2,3]. In the renin–angiotensin system, ACE can catalyze the conversion of angiotensin-I (an inactive decapeptide) into angiotensin-II, with potential vasoconstriction effects; in addition, ACE can inactivate bradykinin that has vasodilatory activity in the kallikrein–kinin system [4]. Undesirable dietary habits are one of the main causes of hypertension [5]. Therefore, the improvement of dietary habits, including the addition of ACE-inhibitory peptides to the diet, is considered a reasonable strategy to lower the blood pressure [6]. Compared with chemically synthesized ACE inhibitors, ACE-inhibitory peptides identified in food proteins have some advantages, as they are economical, can be obtained from various sources, are safe and more easily accepted by consumers [7]. Recently, technologies such as in silico screening, molecular docking and in silico simulated absorption and transport have presented new possibilities for the application of ACE-inhibitory peptides by functional food and pharmaceutical industries [8,9].

However, several challenges must be faced before novel ACE-inhibitory peptides can be used by the food or pharmaceutical industry, which regard their bioavailability, antihypertensive effect, in vivo safety, and stability in different processing conditions [10]. It was demonstrated that the amino acid sequence, especially the *C*-terminal tripeptide, plays a crucial role in the physiological functions of ACE-inhibitory peptides [11]. However, the active sequence of ACE-inhibitory peptides can be degraded by proteases present in the stomach and the intestine such as pepsin, trypsin and dipeptidase, thereby making the peptides inactive in vivo [12]. Moreover, the active sequence of ACE-inhibitory peptides can be modified under some processing conditions including acidic treatment, alkali treatment, heating, fermentation and by the interaction with other food ingredients [13]. Safety is the first requirement for foods. Unsafe factors, especially potential toxicity and allergenicity, hinder the inclusion of bioactive peptides in food [10]. In addition, some physicochemical properties of these peptides, such as isoelectric point, hydrophilicity and hydrophobicity, can also affect their utilization in specific food systems [14]. Therefore, it is very necessary to investigate the safety and physicochemical properties of ACE-inhibitory peptides, as well as their stability in different food processing conditions and during gastrointestinal digestion.

Millet (*Setaria italica*) bran is an abundant and economical plant protein resource because it is rich in protein (8.1–19.6 g/100 g), and its annual yield in China is 480,000 t [15]. Previous studies demonstrated that millet proteins are bioactive, as they may exert anti-inflammatory, hypolipidemic and antioxidant activities [16,17,18]. However, to our best knowledge, little data about millet bran antihypertensive peptides are available. Glutelin-2 accounts for approximately 25 g/100 g of millet proteins [19]. Preliminary experiments for this study demonstrated that the ACE-inhibitory activities of albumin, globulin, prolamin, glutelin-1 and glutelin-2 are 11.47%, 12.67%, 19.00%, 16.41% and 33.56% (at 1 mg/mL), respectively. Millet bran glutelin-2 with high ACE-inhibitory activity and yield can be developed as a potential natural ACE inhibitor. Therefore, the current study focused on the identification, screening, characterization and safety of ACE inhibition peptides from millet bran glutelin-2 hydrolysates using a combined in silico and in vitro strategy. Moreover, the effect of these peptides on ACE structure and stability in various food processing conditions and during gastrointestinal digestion were also studied.

## 2. Materials and Methods

### 2.1. Materials

Millet bran was purchased from Yushe Old Mill, Yushe, China. Trypsin (from bovine pancreas, 5 × 10^4^ U/g), alcalase (from *Bacillus licheniformis*, 2 × 10^5^ U/g), papain (8 × 10^5^ U/g) and pepsin (from porcine stomach, 5 × 10^4^ U/g) were bought from Guangdong Shengwukeji Co., Ltd. (Guangzhou, China). ACE and N-hippuryl-L-histidyl-L-leucine (HHL) were purchased from Sigma (St. Louis, MO, USA). Other chemicals were bought from Lianshi Company (Guangzhou, China).

### 2.2. Preparation of Millet Bran Glutelin-2 Hydrolysates (MBGH)

As per the modified method of Zheng et al. [20], millet bran was crushed and sieved with a 120-mesh sieve. Millet bran powder was deoiled with *N*-hexane (1: 25, *m*/*v*) in triplicate samples. Twenty grams of the defatted millet bran was dispersed in a NaCl solution (250 μmol/L, 400 mL), mixed thoroughly and stirred (180 r/min) at 40 °C for 80 min. After filtration with a filter paper, the residue was collected and dispersed into 0.1 mol/L NaOH (1:10, mg/mL). The mixture was stirred (180 r/min) at 45 °C for 125 min and then filtered with a filter paper. The filtrate was collected and centrifuged at 12,000× *g* for 12 min. The supernatant was dialyzed against deionized water (dH_2_O) with SP132590 dialysis membranes (3500 Da MWCO) at 4 °C for 24 h. The dH_2_O was changed at 4 h intervals. Then, the dialysate solution was dried using an FD1A50 freeze dryer (Kesheng Food Machinery Co., Zhucheng, China) to obtain a millet bran glutelin-2 powder.

Four grams of millet bran glutelin-2 powder was dissolved in 200 mL of distilled water (dH_2_O), and the solution was adjusted to pH 7.8 ± 0.1. Papain (100 U/g) and alcalase (200 U/g) were added and incubated under stirring (180 r/min) in a water bath at 53 °C. The pH value of the reaction solution was maintained at pH 7.8 ± 0.1 by adjusting it with 0.1 mol/L NaOH at 30 min intervals. Two hours later, the pH value was adjusted to pH 6.8, and trypsin (100 U/g) was added. The reaction solution was stirred (180 r/min) at 37 °C for 60 min and then heated at 100 °C for 8 min to inactive the enzymes. After centrifugation at 13,500× *g* for 10 min, the supernatant was dried with the freeze drier. This procedure yielded millet bran glutelin-2 hydrolysates (MBGH). Moreover, the hydrolysis degree of MBGH was determined using the trinitrobenzenesulfonic acid method [21].

### 2.3. ACE Inhibition and Definition of IC_50_ Value

As per the same procedure described by Jimsheena and Gowda [22], the inhibition of ACE was evaluated by comparing the quantity of produced hippuric acid before and after the addition of the peptides. The IC_50_ value was calculated from the regression equation of ACE inhibition percentage for different concentrations of the sample and defined as the concentration of sample inhibiting ACE activity by 50%.

### 2.4. Purification and Identification of ACE-Inhibitory Peptides from MBGH

After ultrafiltration using a membrane with pore size of 0.45 μm, the MBGH (1 mg/mL) was purified with a Sephadex G-15 gel column (Φ1.6 × 100 cm), eluted using distilled water at an elution rate of 2.8 mL/min. The monitored wavenumber was 220 nm, and the effluent fraction was collected every 5 min. The collected fractions were lyophilized, and their ACE inhibition capacity was determined. The fraction with the highest inhibition capacity was further separated using reversed-phase high-performance liquid chromatography (RP-HPLC) with a semi-preparative C_18_ column (Zorbax, 9.4 × 250 mm, Agilent Technologies, Palo Alto, CA, USA). A linear gradient of acetonitrile (5%–35%, in 25 min) was used as mobile phase A, and deionized water containing 0.1% (*v*/*v*) of trifluoroacetate was used as mobile phase B. The flow rate was 2.2 mL/min and was monitored at 220 nm. The subfractions were freeze-dried and used for the determination of ACE inhibition. The subfraction with the highest inhibition capacity was chosen for the analysis of the peptide sequence.

The identification of the peptide sequence was carried out using Ultra-High-Performance Liquid Chromatography (UPLC) coupled with electrospray ionization–mass spectrometry (ESI–MS). A UPLC (U-3000 Series, Thermo Scientific, Waltham, MA, USA) with an InfinityLab Poroshell 120 EC-C_18_ column (80 × 2.0 mm, 1.9 μm, Agilent Technologies, Santa Clara, CA, USA) was performed with a gradient of acetonitrile (5–95%, 0–30 min; 95–5%, 30–35 min) as eluent A and ultrapure water (containing 0.1% formic acid) as eluent B was. The flow rate was 0.3 mL/min. The ESI-MS with a Q Exactive hybrid quadrupole-orbitrap mass spectrometer (Thermo Fisher, Bremen, Germany) was carried out with full MS 35000, ddMS^2^ 17500, AGC target value of 1 e^5^, and mass range of 120–1800 *m/z*. Moreover, the MS data were processed by De Novo™ software (Peak Studio 7.5, Bioinformatics Solutions, Inc., Waterloo, BC, Canada) [23].

### 2.5. In Silico Screening and Synthesis

Peptide sequences identified in the MBGH were analyzed utilizing the databases BIOPEP (http://www.uwm.edu.pl/biochemia/index.php/en/biopep, accessed on 17 December 2021) and AHTPDB (http://crdd.osdd.net/raghava/ahtpdb/, accessed on 17 December 2021) to find sequences of potential ACE inhibitors and of peptides with antihypertensive activity, respectively [24]. If the predicted vector machine software scores (SVMS) of a peptide sequence is more than zero, and the average local confidence (ALC) of a peptide sequence is above 85%, the peptide is acceptable as an ACE inhibitor of with potential antihypertension activity [7,25]. Isoelectric point, hydrophilicity, hydrophobicity and amphiphilicity of the selected peptides were predicted using the database AHTPDB (http://crdd.osdd.net/raghava/ahtpdb/, accessed on 17 December 2021). Moreover, the chemical synthesis of the selected sequence was performed by Qiangyao Biotech *Co*. (Wuxi, China) with a standard solid phase method.

### 2.6. In Silico Security Prediction

Toxicity evaluation of the peptides identified in the MBGH was carried out using the database ToxinPred (http://www.imtech.res.in/raghava/toxinpred/, accessed on 23 December 2021) [25]. In addition, the potential allergenicity of the peptides was assessed using the database AlgPred (http://www.imtech.res.in/raghava/algpred/, accessed on 23 December 2021), with a threshold of −0.4 [26].

### 2.7. Molecular Docking

The docking patterns of ACE and the selected peptide sequences were molecularly visualized with the WYBYL-X2.11 software (X2.11, Tripos International Company, Saint Louis, Missouri, USA) [23]. From the Protein Data Bank (http://www.rcsb.org/pdb/home/home.do, accessed on 9 January 2022), the three-dimensional (3D) crystal structure of ACE (PDB: 1O8A) was downloaded. The T-score (the least required thrust value is 6.0) was the predominant indicator used to select the docking patterns between the peptide and ACE [27]. Additionally, the C-score, indicating the number and distance of the hydrogen bonds formed between the active sites of ACE and the peptide sequence, was also recorded.

### 2.8. Stability Profiles under Different Processing Conditions

#### 2.8.1. Thermal Stability Profiles

As per the method described by Zheng et al. [13], the synthesized peptide sequences identified in the MBGH were dissolved in dH_2_O (100 μg/mL, pH 7.0) and then subjected to two thermal regimens. (i) The peptide solution was heated at different temperatures to simulate pasteurization conditions, including heating at 63 °C for 0.5 h; 69 °C for 0.5 h; 72 °C for 15 s; 75 °C for 10 min; 80 °C for 25 s; and 100 °C for 12 min. (ii) The peptide solution was heated at 100 °C for 10, 20, 30, 40 and 50 min. After each thermal processing, the peptide solution was cooled to room temperature, and its ACE inhibitory activity was determined. Untreated peptides were used as a control.

#### 2.8.2. pH Stability Profiles

According to the method described by Chai et al. [28] with slight modifications, the synthesized peptide sequences identified in the MBGH were dissolved in dH_2_O (100 μg/mL), and the solutions were then separately adjusted to different pH values (pH 2–10). After incubation at 37 °C for 10 min, these solutions were all adjusted to pH 7.0. After that, these peptide solutions were used for the determination of their ACE inhibitory activity, using an untreated sample for comparison.

#### 2.8.3. Effects of Different Metal Ions on Peptide Stability

Peptide stability in the presence of different metal ions was evaluated according to the method of Zheng et al. [13]. Briefly, the peptides identified in the MBGH were dissolved in dH_2_O (1 mg/mL). An aliquot of each peptide solution (100 μL) was subjected to treatment with NaCl, KCl, MgSO_4_, ZnSO_4_ and FeCl_3_ (5 mmol/L, 100 μL). After incubation at 37 °C in a stirring bath (140 r/min) for 15 min, 50 μL of the reaction solution was taken, and its ACE inhibitory activity was determined. The remaining reaction solution was mixed with the same volume of ethanol (approximately 150 μL) and then centrifuged at 12,000× *g* for 10 min. The precipitate was collected, lyophilized and then mixed with dry KBr (1:50 *m*/*m*). The mixed powder was ground, pelleted and analyzed using a 660-IR FTIR spectrometer (Varian, Palo Alto, CA, USA) in a scanning range from 400 to 4000 cm^−1^ [13]. Untreated peptides were used for comparison.

### 2.9. Stability Profiles during Simulated Gastrointestinal Digestion

Mixtures of trypsin (0.45 g), pig bile salt (3 g) and NaHCO_3_ (6.25 g) dissolved in 100 mL of dH_2_O (pH 6.8) were used to simulate the intestinal fluid, whereas pepsin (40 mg) and NaCl (0.877 g) dissolved in 100 mL of dH_2_O (pH 2.0) were used to simulate the gastric fluid (Sun et al., 2021) [29]. Peptide solutions (dissolved in ultrapure water, 1 mg/mL) were incubated at 37 °C in a stirring bath (140 r/min) for 15 min, and then the simulated gastric fluid was added (gastric fluid: peptide solution = 1: 10 *v*/*v*), and the mixture was further incubated at 37 °C (140 r/min) for 90 min. Then, the reaction solution was adjusted to pH 6.8 with Na_2_HPO_4_. The simulated intestinal fluid (the fluid: peptide solution = 1: 10, *v*/*v*) was added, and the mixture was stirred (140 r/min) at 37 °C for 180 min. All reaction solutions were heated at 100 °C for 8 min to inactivate the enzymes. The ACE inhibition activities of the peptides before and after digestion were separately determined to evaluate their stability.

### 2.10. Data Analysis

Data were analyzed, and the results are expressed as mean ± standard errors (*n* ≥ 3) (SPSS Version 16.0 software, Chicago, IL, USA). One-way ANOVA was used to analyze the variance at a significance level of *p* < 0.05.

## 3. Results and Discussion

### 3.1. Isolation of ACE-Inhibitory Peptides from MBGH

**Figure 1:**
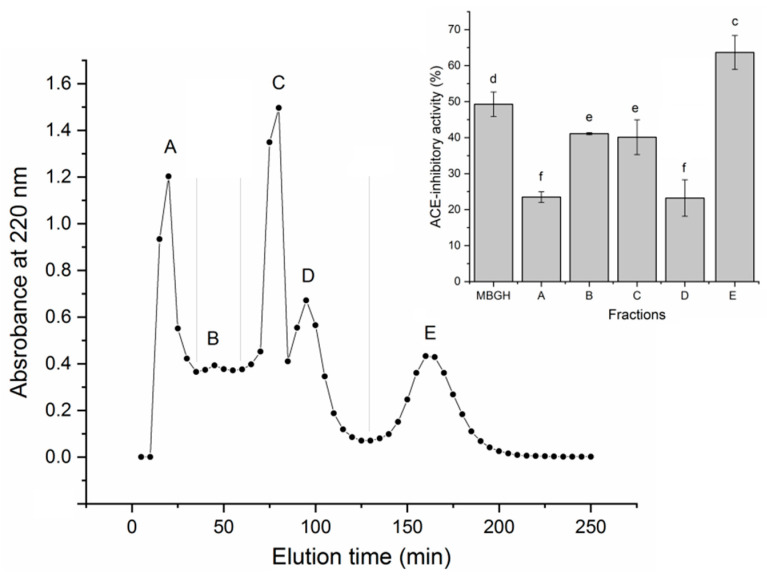
Sephadex G-15 gel chromatograms of the millet bran glutelin-2 hydrolysate (MBGH) and ACE-inhibitory activity of its subfractions (A–E). Various lowercase letters (c–f) on the bars indicate significant differences (*p* < 0.05).

**Figure 2:**
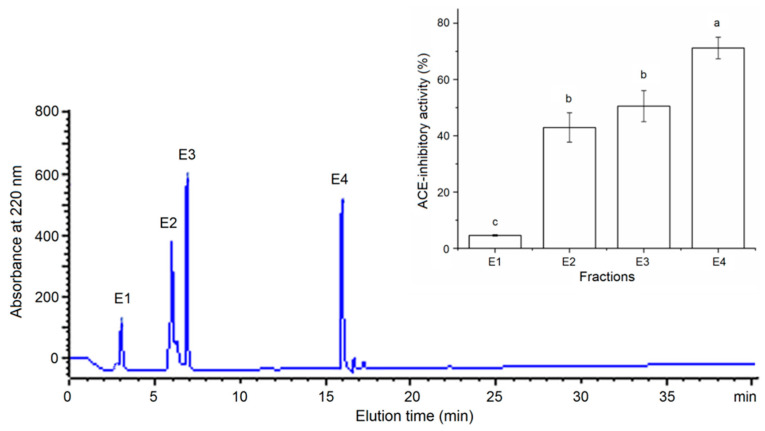
Purification spectrum of the MBGH-E fraction determined by semi-preparative RP-HPLC and ACE inhibition capacity of each subfraction. E1, E2, E3 and E4 above the peaks indicate the subfractions separated from the MBGH-E fraction. Various lowercase letters (a–c) above the bars indicate that the differences are significant (*p* < 0.05).

The extraction ratio of millet bran glutelin-2 was 6.94 g/100 g millet bran, consistently with the report of Fu et al. [19]. The ACE inhibition capacity of millet bran glutelin-2 was 33.56% (at 1 mg/mL). After digestion by alcalase, papain and trypsin, the hydrolysis degree of millet bran glutelin-2 hydrolysates (MBGH) was 19.33% ± 1.18%, and the ACE-inhibitory activity of MBGH was 49.28% ± 3.38% (at 1 mg/mL). Alcalase, papain and trypsin are widely used in the preparation of antihypertensive peptides because they preferentially hydrolyze peptide bonds linking aromatic amino acid residues, Lys or Arg residues, which are mainly responsible for the ACE-inhibitory activity of peptides [4]. As shown in Figure 1, through Sephadex G-15 gel column chromatography, the MBGH was separated into MBGH-A, MBGH-B, MBGH-C, MBGH-D and MBGH-E fractions. Because MBGH-E showed greater ACE inhibition capacity than the other subfractions (*p* < 0.05), it was further purified using RP-HPLC with a semi-preparative C_18_ column. As shown in Figure 2, four main peaks (MBGH-E1, MBGH-E2, MBGH-E3 and MBGH-E4) appeared after the purification of MBGH-E with RP-HPLC. MBGH-E4 was chosen for amino acid sequencing with UPLC–ESI–MS/MS because its ACE inhibitory activity was the highest (71.19% ± 3.85%, at 1 mg/mL).

The recent development of technologies such as in silico prediction of bioactivity and safety of peptides, molecular docking and in silico simulated absorption and transport in vitro has made the identification and selection procedures of peptides faster, easier and more precise [8]. However, primary isolation and purification are still necessary, since they can exclude most of the not-targeted peptides and improve the accuracy of the identification [30,31]. Thus, a combined strategy including a classic purification procedure (Sephadex gel and PR-HPLC chromatography) and in silico screening was utilized in the current study.

### 3.2. Peptide Identification from MBGH-E4 and In Silico Screening

**Figure 3:**
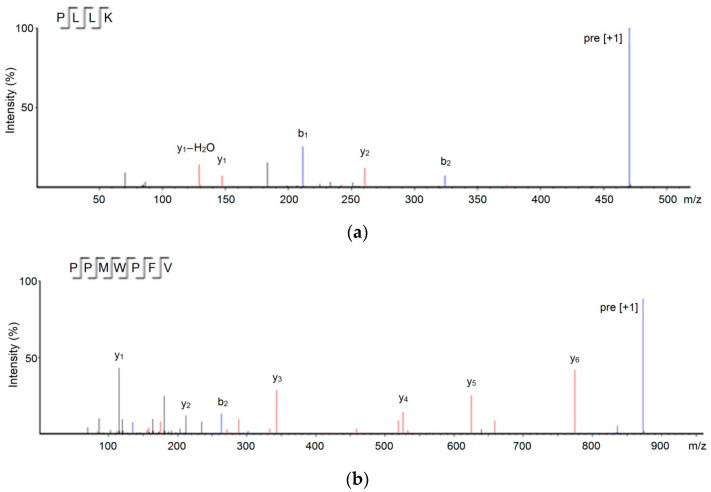
ESI–MS/MS spectra of PLLK (**a**) and PPMWPFV (**b**) identified in millet bran glutelin-2 hydrolysates.

Based on the results of the UPLC-ESI-MS/MS analysis, regarding, especially fragment information and molecular weight, four oligopeptides of 4–9 amino acid residues were identified in MBGH-E4 (Table 1). The in silico screening of these peptide sequences is shown in Table 1. Since the average local confidence (ALC) was greater than 85%, the peptides Pro-Leu-Leu-Lys (469.66 Da) and Pro-Pro-Met-Trp-Pro-Phe-Val (873.18 Da) were demonstrated to have ACE inhibitory activity [25]. Furthermore, PLLK and PPMWPFV were also predicted to have high potential antihypertensive capacity, because their vector machine software scores (SVMS) were higher than 0.9 [7]. These sequences were chemically synthesized, and their ESI–MS/MS spectra are shown in Figure 3.

**Figure 4:**
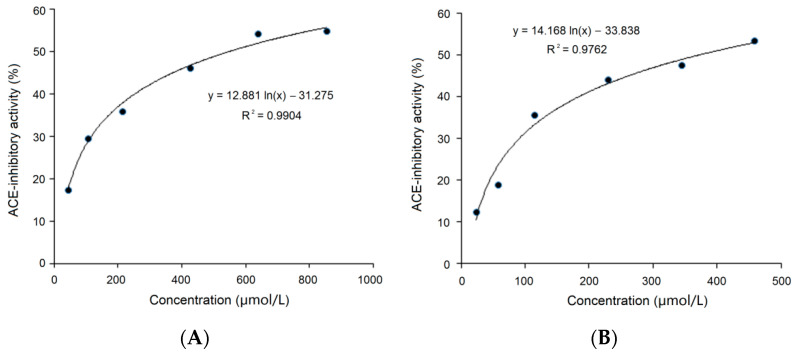
Regression analysis of the ACE inhibitory activity of PLLK (**A**) and PPMWPFV (**B**).

**Figure 5:**
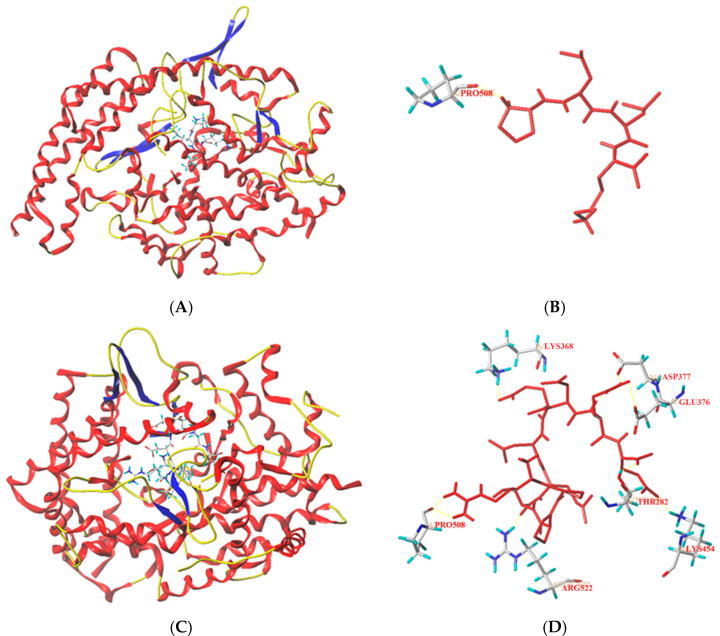
General overview and local overview of the best-ranked binding patterns of PLLK (**A**,**B**) and PPMWPFV (**C**,**D**) docking at ACE (PDB: 1O8A).

On the basis of the regression equations (y = 12.881 ln(x)–31.275, *R^2^* = 0.99) and (y = 14.168 ln(x)–33.838, *R^2^* = 0.9762), as shown in Figure 4A,B, the IC_50_ values of PLLK and PPMWPFV were calculated to be 549.87 μmol/L and 364.62 μmol/L, respectively. Obviously, PLLK was identified as a tetrapeptide with a high content of Leu (a branched amino acid), while PPMWPFV was identified as a heptapeptide with the branched amino acid Val, the aromatic amino acid Phe and a Pro residue in the *C*-terminal tripeptide. Previous studies referring to structure–activity relationships demonstrated that the ACE inhibition capacity of peptides is mainly dependent on a tripeptide sequence of amino acids especially at the *C*-terminal [12]. In particular, aromatic amino acids (Phe, Trp and Tyr), branched amino acids (Leu, Ile and Val) and Pro in *C*-terminal tripeptides are mainly responsible for the high ACE-inhibitory activity of peptides, because these amino acids can tightly bind to active sites in ACE [7,32]. Moreover, an increasing number of studies have demonstrated that a Lys residue in a peptide sequence could tightly bind to the key site S1 of ACE, thereby remarkably improving the inhibition capacity of peptides towards ACE [33]. Therefore, the peptide sequence characteristics of PLLK and PPMWPFV appear to be responsible for their relatively high ACE inhibitory activity. In addition, PLLK (469.66 Da) showed a lower ACE inhibitory activity than the peptides SSYYPFK, derived from oat naked glutelin-2 (890.4 Da, IC_50_: 91.82 μmol/L), and NMAINPSKENLCSTFCK, identified in casein (IC_50_: 129.07 μmol/L). This is inconsistent with reports that the ACE inhibitory ability of a peptide is negatively correlated with the peptide mass [11,34,35]. The main reason of this discrepancy is perhaps the use of different inhibition models of ACE and the different binding power to ACE, as shown in Figure 5. In addition, the IC_50_ values of PLLK and PPMWPFV were much higher than that of Captopril (an excellent antihypertensive drug with an IC_50_ value of 0.14 μmol/L) [20], indicating that their dose required for a therapeutic effect is likely to be relatively high. This suggests that these peptides may have an auxiliary hypotensive effect.

### 3.3. In Silico Prediction of Physicochemical Properties and Potential Safety

Some physicochemical properties of PLLK and PPMWPFV were predicted using the BIOPEP databases [24]. As shown in Table 1, the hydrophobicity of PLLK and PPMWPFV is 0.22 and 0.53, respectively, consistent with their high content of hydrophobic amino acid residues. Their high amphiphilicity (0.92 and 0.99) suggested that PLLK and PPMWPFV have relatively high solubility in both polar food systems and non-polar food systems [10]. Moreover, the isoelectric point of PLLK and PPMWPFV is 9.11 and 5.88, respectively, indicating that it should be avoided to use them in food systems with these pH values [13].

In silico prediction provides fast and low-cost information on the safety of novel peptides. The result predicted by the database ToxinPred demonstrated that PLLK and PPMWPFV are not toxic (Table 1). The allergenicity of these peptides was not predicted because only peptides with more than 12 amino acids can be analyzed in AlgPred (www.imtech.res.in/raghava/algpred/, accessed on 23 December 2021). A previous study reported that oligopeptides with a smalle mass derived from foods are less allergenic in comparison with proteins having a large mass, because they usually do not contain complete epitopes [10]. Moreover, since some ACE inhibitors have been shown to affect bradykinin metabolism and cause cough [4], further study in vivo is still needed to clarify the safety of these peptides.

### 3.4. Molecular Docking Analysis

Peptides can bind to key binding pockets in ACE (S1, S_1_′, and S_2_′) and interfere with the binding of ACE to its ligands (angiotensin-I or bradykinin), thereby showing competitive inhibition [32]. The results of molecular docking revealed that PPMWPFV can bind to seven active sites in ACE (LYS368, ASP377, GLU376, THR282, LYS454, ARG522 and PRO508P) and form eight short hydrogen bonds (Figure 5 and Table 2), suggesting that PPMWPFV has a relatively strong binding affinity for ACE [27]. This is the main reason for its relatively high T-Score (9.93) and inhibition activity (364.62 μmol/L). On the other hand, PLLK also showed a high T-Score (11.76), although it can bind to only one active site of ACE (PRO508) and form two hydrogen bonds. The main reason is perhaps that the distance of these hydrogen bonds is short (Table 2), since a high T-Score or a short distance between hydrogen bonds indicates a strong coordination between peptides and ACE [36]. Moreover, the lower hydrogen bond number is responsible for the lower ACE inhibitory activity of PLLK compared to PPMWPFY [7]. In addition, the active sites with which PPLK and PPMWPFV interact are not in the key binding pockets of ACE (S1, S_1_′ and S_2_′), so both PPLK and PPMWPFV are non-competitive inhibitors of ACE [32]. The non-competitive inhibition is the main reason why PPLK and PPMWPFV showed a weaker ACE inhibitory capacity than the peptides NMAINPSKENLCSTFCK identified in casein (a competitive inhibitor of ACE, IC_50_: 129.07 μmol/L) and SSYYPFK derived from oat naked globulin (a competitive inhibitor, IC_50_: 91.82 μmol/L) [34,35].

### 3.5. Stability under Different Thermal Treatments and pH Values

**Figure 6:**
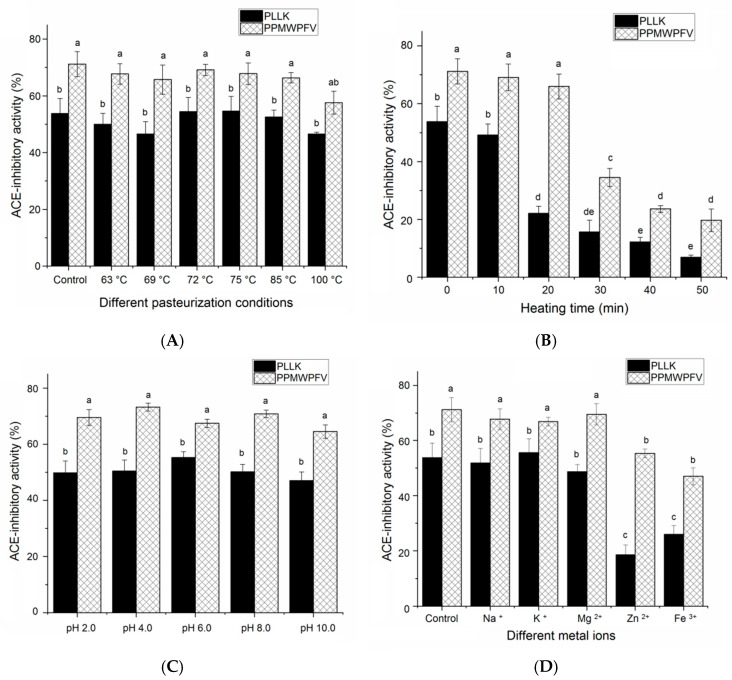
Stability of PLLK and PPMWPFV (**A**) in different pasteurization conditions: heating at 63 °C, 0.5 h; 69 °C, 0 h; 72 °C, 15 s; 75 °C, 10 min; 80 °C, 25 s; and 100 °C, 12 min; (**B**) incubation at 100 °C for 10–50 min; (**C**) at various pH values; (**D**) addition of various metal ions. Controls in each figure are untreated samples. Different lowercase letters (a–e) above the bars indicate the difference is significant (*p* < 0.05).

Stability is one paramount factor determining the compatibility of peptides with different food systems and their bioavailability in vivo [28]. Heat treatments such as pasteurization and boiling are common processing techniques in the food industry. Obviously, the ACE inhibition capacities of PPLK and PPMWPFV were relatively stable in different pasteurization conditions (Figure 6A). These conditions included thermal treatments at 63 °C for 0.5 h, 69 °C for 0.5 h, 72 °C for 15 s, 75 °C for 10 min and 80 °C for 25 s. A reduction in the ACE inhibition capacity of both PLLK and PPMWPFV was observed after treatment at 100 °C for 12 min, but it was insignificant (*p* > 0.05). Moreover, the result in Figure 6B demonstrated that PPLK and PPMWPFV showed stable ACE inhibition activity during heating at 100 °C for 10–20 min. However, the ACE inhibition capacity of both PPLK and PPMWPFV was dramatically decreased by a heat treatment at 100 °C for more than 30 min (*p* < 0.05), indicating that they are unstable when subjected for a long time to a high temperature. The structure of peptides can be destroyed at a high temperature, and their bioactivity will be lost [10].

The ACE-inhibitory activities of PPLK and PPMWPFV were relatively stable in a pH value range of 2.0–10.0 (Figure 6C). It is well recognized that the structure of a protein tends to shrink at the isoelectric point. This tendency probably influences the docking modes of ACE and peptides [14]. However, PLLK did not show an obviously different ACE inhibition capacity at pH 10.0 (near its isoelectric point of pH 9.11, Table 1) with respect to other pH values (*p* > 0.05). A similar trend was observed for PPMWPFV. The main reason of this behavior is that the effect of the isoelectric point on oligopeptides is smaller than that on proteins with a large molecule weight [2].

### 3.6. Effects of Different Metal Ions on the Stability

#### 3.6.1. Stability Profiles

Previous studies have found that the addition of metal ions can affect the capacity for ACE inhibition of peptides in two ways: (i) by changing the polarity of the microenvironment around the catalytic sites in the active center of ACE [7]; (ii) by inducing the formation of a peptide–metal chelate [29]. Both mechanisms can influence the binding patterns of ACE and peptides, resulting in a change in the peptides’ ACE inhibitory capacity. Moreover, the zinc ion is the key prosthetic group of ACE [27]. The addition of zinc ions has been demonstrated to have an effect on the ACE inhibition capacity of peptides identified in camellia’s proteins [13]. After treatment with Na^+^, K^+^ or Mg^2+^, the ACE inhibitory activity of PLLK or PPMWPFV was not significantly different compared to that of the untreated peptides (Figure 6C) (*p* > 0.05), suggesting that the addition of Na^2+^, K^+^ and Mg^2+^ had no obvious effect on the ability of PLLK and PPMWPFV to inhibit ACE. In contrast, both PLLK and PPMWPFV exhibited a much lower ACE inhibitory activity after the addition of Zn^2+^ or Fe^3+^ (*p* < 0.05), indicating that the ACE inhibitory activity of PLLK and PPMWPFV were susceptible to high concentrations of Zn^2+^ or Fe^3+^.

#### 3.6.2. Fourier-Infrared Spectroscopy Analysis

**Figure 7:**
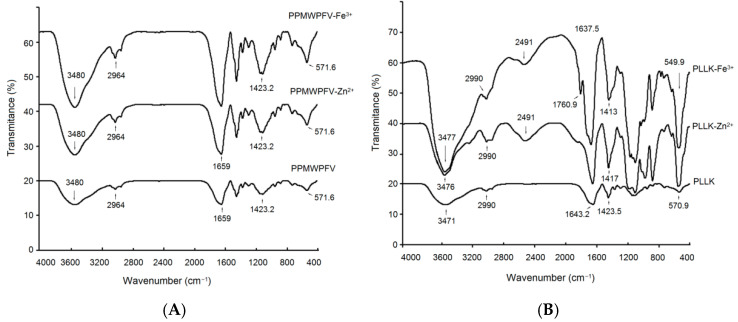
Fourier-transformed infrared spectra of PPMWPFV (**A**) and PLLK (**B**) after treatment with Zn^2+^ and Fe^3+^. PLLK–Zn^2+^, PLLK–Fe^3+^, PPMWPFV–Zn^2+^ and PPMWPFV–Fe^3+^ are PLLK and PPMWPFV after reaction with ZnSO_4_ and FeCl_3_ (5 mmol/L), respectively.

The FT-IR spectrum of PPMWPFV treated with Zn^2+^ or Fe^3+^ showed no obvious differences compared with that of untreated PPMWPFV (*p* > 0.05) (Figure 7A), demonstrating that the metal treatments did not cause the formation of PPMWPFV–iron chelate or PPMWPFV–zinc chelate. In contrast, an obvious difference was found in the FT-IR spectrum of PLLK after treatment with Zn^2+^ or Fe^3+^ (*p* < 0.05, Figure 7B). Compared with the FT-IR spectrum of untreated PLLK, the adsorption peak at 3471 cm^−1^, corresponding to the stretching vibration of −N–H, shifted to 3476 cm^−1^ and 3477 cm^−1^ after the addition of Zn^2+^ and Fe^3+^, respectively (Figure 7B) [37]. Moreover, the peak at 570.90 cm^−1^, representative of the stretching of amide IV in PLLK, separately shifted to 551.7 cm^−1^ and 549.9 cm^−1^ after the addition of Zn^2+^ and Fe^3+^ [29]. These results indicate that the N-terminal amino groups (–NH_3_) of PLLK are involved in the chelation of Zn^2+^ and Fe^3+^ [38]. The adsorption peak at 1423.5 cm^−1^ (representing the strength of the −C=O bond of amide I) in the spectrum of untreated PLLK moved to 1417 cm^−1^ and 1413 cm^−1^ after treatment with Zn^2+^ and Fe^3+^, respectively [39]. Moreover, a new peak appeared at 1760.9 cm^−1^ owing to the deformation vibration of the carboxyl group in the spectrum of PLLK treated with Fe^3+^, suggesting the formation of –COO–Fe [29].

Overall, the results shown in Figure 6C and Figure 7B demonstrated the formation of PLLK–iron chelates and PLLK–zinc chelates. Chelation with Zn^2+^ or Fe^3+^ can induce changes in the structure of PLLK, thereby reducing its ACE inhibitory activity. In addition, treatment with Fe^3+^ or Zn^2+^ may change the polarity of the microenvironment around the active center of ACE, leading to a decrease in the ACE inhibitory activity of PPMWPFV [14].

### 3.7. Resistance to Gastrointestinal Digestion

**Figure 8:**
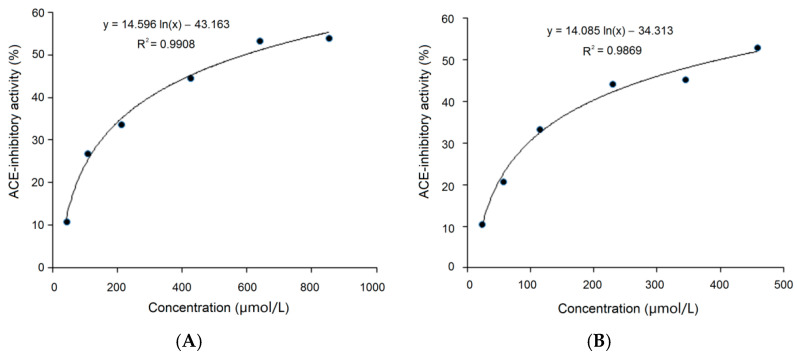
Regression analysis of the ACE inhibitory activity of PLLK (**A**) and PPMWPFV (**B**) after simulated gastrointestinal digestion.

ACE-inhibitory peptides should have sufficient resistance to digestion by proteases existing in the gastrointestinal tract to exert antihypertension activity in the body [37]. As shown in Figure 8A, on the basis of the regression equation y = 14.596 ln(x)–43.163, the IC_50_ value of PLLK after digestion was calculated to be 591.57 μmol/L (Table 1). This value was higher, though not significantly, than that of untreated PLLK (364.62 μmol/L, Figure 4A) (*p* > 0.05). Moreover, the IC_50_ value of PPMWPFV after digestion was 397.83 μmol/L, as calculated from the regression equation y = 14.085 ln(x)–34.313 (Figure 8B), and was not significantly different compared to that of the untreated PPMWPFV (364.62 μmol/L, Figure 4B). These results indicated that both PLLK and PPMWPFV were relatively stable during the simulated gastrointestinal digestion. PPMWPFV contains Tyr and Phe residues but exhibited a relatively high stability against gastrointestinal digestion, which seems to be inconsistent with the report that peptides rich in aromatic amino acids (Tyr, Phe and Trp) are susceptible to gastrointestinal digestion [29], because pepsin and trypsin in the gastric and the intestinal fluids preferentially hydrolyze amide bonds involving aromatic amino acid residues [12]. The main reason of our observations is perhaps the high proline content of PPMWPFV. Proline has been demonstrated to have a high water-holding capacity and can confer plants excellent resistance to drought and heat [40]. An increasing number of studies found that a Pro residue near aromatic amino acids can effectively improve the resistance of a peptide to digestion by pepsin and trypsin [32]. Therefore, the Pro residue in PLLK and PPMWPFV may contribute to peptide stability during gastrointestinal digestion. Proline-rich peptides including ELHPQ, LHPQ and KPVPR, identified in canary seeds, and SSYYPFK, identified in naked oat, also exhibited excellent stability during gastrointestinal digestion [7,34]. Moreover, the Lys (cationic amino acid) residue at the *C*-terminus of PLLK and the branched amino acid residues (Leu and Val) existing in PLLK and PPMWPFV were also demonstrated to be helpful for the stability of oligopeptides during gastrointestinal digestion [10,29]. In addition, the application of trypsin during proteolysis could enhance the resistance of peptides to gastrointestinal hydrolysis [34]. However, the influence of simulated gastrointestinal digestion on the amino acid sequences of PLLK and PPMWPFV should be studied in a future work.

## 4. Conclusions

Two novel ACE-inhibitory peptides, PLLK (IC_50_: 549.87 μmol/L) and PPMWPFV (IC_50_: 364.62 μmol/L), without potential toxicity, were identified in millet bran glutelin-2 hydrolysates. PLLK and PPMWPFV can bind to one and seven binding sites of ACE, (but not to the key binding sites), respectively, through hydrogen bonds of a short distance. Both PLLK and PPMWPFV were resistant to different pH values (2.0–10.0), pasteurization conditions, and addition of Na^+^, Mg^2+^ or K^+^. Moreover, PLLK and PPMWPFV showed a relatively high stability during simulated gastrointestinal digestion. However, these peptides were unstable when heated at 100 °C for more than 30 min and when treated with Fe^3+^ or Zn^2+^. The addition of Fe^3+^ or Zn^2+^ could induce the formation of PLLK–iron chelate or PLLK–zinc chelate. These results indicated that PLLK and PPMWPFV derived from millet bran could be used as ingredients for antihypertensive foods. However, their bioavailability, plasma half-life, antihypertensive effect in vivo and other experimental indicators should be investigated in further work.

## Figures and Tables

**Table 1:** Amino acid sequences obtained from UPLC–ESI–MS/MS analysis, ACE inhibition capacity, in silico prediction of toxicity, allergenicity and physicochemical properties of the peptides identified in millet bran glutelin-2 hydrolysates.

Peptide Sequence	PLLK	SGGRGGFGGG	NDFAGF	PPMWPFV
Mass (Da)	469.66	807.97	669.76	873.18
ALC (%)	90	76	70	94
SVMS	0.99	−0.10	−0.94	0.97
Prediction	AHT	Non-AHT	Non-AHT	AHT
Hydrophilicity	−0.15	0.08	0.00	−1.24
Amphiphilicity	0.92	0.25	0.00	0.99
Hydrophobicity	0.53	−0.03	0.05	0.22
Isoelectric point	9.11	10.11	3.80	5.88
IC_50_ (μmol/L)	549.87	ND	ND	364.62
IC_50_ (μmol/L) after gastrointestinal digestion (μmol/L)	591.57	ND	ND	397.83
Toxicity	Non-Toxin	Non-Toxin	Non-Toxin	Non-Toxin
Allergenicity	ND	ND	ND	ND

The BIOPEP database was used for the calculation of the average local confidence (ALC); AHT: antihypertension; SVMS: vector machine software score. The AHTPDB database was employed to predict the physicochemical properties. The potential toxicity and allergenicity were predicted using the databases ToxinPred (www.imtech.res.in/raghava/toxinpred/, accessed on 23 December 2021) and AlgPred (www.imtech.res.in/raghava/algpred/, accessed on 23 December 2021), respectively. ND: not measured.

**Table 2:** Docking scores and hydrogen bonds observed between ACE and peptides PLLK or PPMWPFV from the molecular docking simulation.

Ligand	T-Score	C-Score	Hydrogen Bonds Number	Distance (Å)
PLLK	11.76	5.00	2	PRO508: 1.84, 1.92
PPMWPFV	9.93	5.00	8	LYS368: 2.44; ASP377: 2.73; GLU376: 2.42; THR282: 2.37; LYS454: 2.04; ARG522: 2.73; PRO508: 2.04, 1.95

## Data Availability

Data is contained within the article.
